# Google Street View-Derived Neighborhood Characteristics in California Associated with Coronary Heart Disease, Hypertension, Diabetes

**DOI:** 10.3390/ijerph181910428

**Published:** 2021-10-03

**Authors:** Thu T. Nguyen, Quynh C. Nguyen, Anna D. Rubinsky, Tolga Tasdizen, Amir Hossein Nazem Deligani, Pallavi Dwivedi, Ross Whitaker, Jessica D. Fields, Mindy C. DeRouen, Heran Mane, Courtney R. Lyles, Kim D. Brunisholz, Kirsten Bibbins-Domingo

**Affiliations:** 1 Department of Epidemiology and Biostatistics, School of Public Health, University of Maryland, College Park, MD 20742, USA; dwvdpallavi@gmail.com (P.D.); hmane@umd.edu (H.M.); 2 Department of Family and Community Medicine, University of California San Francisco, San Francisco, CA 94110, USA; 3 Department of Epidemiology and Biostatistics, University of California San Francisco, San Francisco, CA 94158, USA; Anna.Rubinsky@ucsf.edu (A.D.R.); Jessica.Fields@ucsf.edu (J.D.F.); Mindy.Hebert-Derouen@ucsf.edu (M.C.D.); Courtney.Lyles@ucsf.edu (C.R.L.); kirsten.bibbins-domingo@ucsf.edu (K.B.-D.); 4 Department of Electrical and Computer Engineering, Scientific Computing and Imaging Institute, University of Utah, Salt Lake City, UT 84112, USA; tolga@sci.utah.edu; 5 School of Computing, Scientific Computing and Imaging Institute, University of Utah, Salt Lake City, UT 84112, USA; a.nazem93@gmail.com (A.H.N.D.); whitaker@cs.utah.edu (R.W.); 6 UCSF Center for Vulnerable Populations, Zuckerberg San Francisco General Hospital, San Francisco, CA 94110, USA; 7 Division of General Internal Medicine, Department of Medicine, University of California San Francisco, San Francisco, CA 94158, USA; 8 Intermountain Healthcare Delivery Institute, Intermountain Healthcare, Salt Lake City, UT 84107, USA; Kim.Brunisholz@imail.org

**Keywords:** built environment, Google Street View, chronic conditions, computer vision, electronic health records

## Abstract

Characteristics of the neighborhood built environment influence health and health behavior. Google Street View (GSV) images may facilitate measures of the neighborhood environment that are meaningful, practical, and adaptable to any geographic boundary. We used GSV images and computer vision to characterize neighborhood environments (green streets, visible utility wires, and dilapidated buildings) and examined cross-sectional associations with chronic health outcomes among patients from the University of California, San Francisco Health system with outpatient visits from 2015 to 2017. Logistic regression models were adjusted for patient age, sex, marital status, race/ethnicity, insurance status, English as preferred language, assignment of a primary care provider, and neighborhood socioeconomic status of the census tract in which the patient resided. Among 214,163 patients residing in California, those living in communities in the highest tertile of green streets had 16–29% lower prevalence of coronary artery disease, hypertension, and diabetes compared to those living in communities in the lowest tertile. Conversely, a higher presence of visible utility wires overhead was associated with 10–26% more coronary artery disease and hypertension, and a higher presence of dilapidated buildings was associated with 12–20% greater prevalence of coronary artery disease, hypertension, and diabetes. GSV images and computer vision models can be used to understand contextual factors influencing patient health outcomes and inform structural and place-based interventions to promote population health.

## 1. Introduction

Neighborhood environments influence the ability of individuals and communities to access necessary resources for achieving and maintaining good health. Neighborhood attributes have been linked to a broad array of health outcomes including mortality [1,2,3], life expectancy [4], mental health [5], self-rated health, obesity [6,7], and diabetes [8]. Importantly, neighborhood resources and risks are not randomly distributed. Historical and contemporary structural racism and inequality has led to racial residential segregation and systemic disinvestment of resultant neighborhoods where minoritized racial/ethnic populations are concentrated [9]. This has resulted in these populations having disproportionate exposure to adverse neighborhood conditions [7,10,11], thereby increasing health inequities. In a recent study of Seattle, San Diego, and Baltimore, neighborhoods with lower socioeconomic status (SES) and higher proportions of racial/ethnic minorities had poorer aesthetics (e.g., dilapidated buildings, graffiti, broken windows, and litter). Conversely, neighborhoods with higher socioeconomic status and lower proportions of racial/ethnic minorities had better pedestrian amenities in terms of sidewalks, crosswalks, and intersection control features [10].

Green spaces have been associated with physical activity, social cohesion, social capital, and stress reduction [11,12,13]. Green space has been hypothesized to impact health through multiple mechanisms. Green space may increase positive perceptions of environment quality and provide incentives for physical activity and encourage use of outdoor neighborhood spaces and social activities within these outdoor spaces [14,15]. Physical disorder has been found to be related to worse health outcomes via psychosocial processes (depressive symptomatology, psychological and physiological distress, and perceived mastery) and lowered physical activity [16]. Physical disorder has also been related to lower sleep quality and self-rated health [17]. Dilapidated buildings including dilapidated housing may increase exposure to lead, pests, air pollutants, and contaminants [18,19]. Similarly, visible wires indicate physical disorder in a neighborhood and have been associated with worse health outcomes [20]. The literature on visible wires is in its infancy and more of this work has been done abroad, for instance in Rio de Janeiro, where the wires represent both an unattractive presence and a possible electrocution/electrical fire risk [21]. In the United States, visible wires have mainly a visual impact on the landscape but could serve as an indicator of physical disorder that might have links to important health outcomes. Visible utility wires hanging overhead are visually striking and may impact residents’ aesthetic sense of their environment, alter perceptions of safety, and influence both mental health (by increasing stress levels) and physical health (by discouraging walking). Dilapidated buildings and visible wires are indicators of disinvestment and may increase safety concerns.

However, the overall lack of neighborhood data on the built environment prevents greater understanding of the impact of neighborhood conditions on health. Neighborhood studies are often conducted on a small scale and include only a few neighborhoods or cities given the typically time- and resource-intensive nature of neighborhood audits. Google Street View (GSV) images provide an alternative to field assessments and would enable neighborhood studies incorporating larger geographies. Globally, GSV image coverage is more complete for some regions than others, with cities in developed nations having near-complete coverage while some low- and middle-income countries in Africa, Southeast Asia, and South America have no GSV imagery at all [22]. Although precise coverage metrics are not available, the U.S. has near-complete coverage [23,24].

GSV images offer a unique lens into the local built environment with ground-level views not possible with other data sources such as satellite data, and provide flexibility to extract a variety of built environment features from one data source. For example, the same image can be used to identify specific built environment features of interest such as crosswalks, commercial buildings, highways, and grasslands. Previous research utilizing GSV has found it to be consistent with field assessments [25,26,27]. Our study contributes to the nascent body of literature utilizing GSV images to implement virtual neighborhood audits for neighborhood features such as walkability [28], physical disorder [26], retail alcohol stores [29], urban greenery [30], pedestrian count [31], and visual enclosure (i.e., the proportion of the sky visible from a point on the street) [32]—which are measures related to walkability.

### Study Aims and Hypotheses

This study examines whether we observe an association between GSV-derived built environment characteristics and health even after adjusting for available measures of individual SES as well as for neighborhood SES. Higher SES individuals may choose to live in neighborhoods with more greenery and street landscaping, better maintained buildings, and amenities. However, within neighborhoods, there is still variation in individual SES. The built environment is related but is conceptually distinct from SES. The built environment is also modifiable. Identifying particular characteristics of the neighborhood built environment that are associated with health outcomes may facilitate levers for improving population health.

In this study, we collected and analyzed GSV images to gain a street level view of neighborhood environments. We investigate whether variation in the prevalence of specific attributes of the neighborhood built environment derived from GSV images (i.e., green space, visible utility wires, and dilapidated buildings) is associated with individual-level patient health outcomes as extracted from electronic medical records. We assessed heart disease, hypertension, and diabetes among California residents receiving outpatient care at the University of California, San Francisco (UCSF) Health System. These health outcomes were chosen given the previous literature connecting built environments to chronic conditions. We hypothesized that green space would be associated with lower prevalence of each chronic condition, and that visible utility wires and dilapidated building conditions (indicators of physical disorder) would be related to a higher prevalence of each chronic condition, independent of patient-level sociodemographic characteristics and neighborhood socioeconomic status. The study demonstrates how GSV images and computer vision models provide valuable opportunities for assessing the built environment. The findings enable us to gain a better understanding of the association between built environment features and health outcomes, and can help to inform health and healthcare organizations of the influence of contextual characteristics on their patients’ health risks.

## 2. Materials and Methods

### 2.1. Google Street View Image Data

Using Google Street View Image API, we collected the most recent GSV images available for street intersections and for sampled locations 50 m apart along all the primary and secondary roads. For each location, we obtained four GSV images (west, east, north, and south) to capture the neighborhood built environment. In total, there were 2,267,109 images obtained in November 2019 from the census tracts represented in the UCSF patient data. The mean date of the image was 2013 (range: 2007–2019). This study was approved by the University of California, San Francisco Institutional Review Board (IRB #17-22277).

Currently, Google’s Street View API only allows users to download the most recent images by default. However, other researchers have built a module for obtaining historical images by retrieving panorama IDs from Google Street View based on desired GPS coordinates and then using these IDs to retrieve available historical images [33]. Nonetheless, because of the broad date range of current GSV images and because residential histories are not available in the UCSF’s electronic health records, the current study focuses on the most recent residential address for any given patient and the corresponding GSV images.

### 2.2. Neighborhood Definitions

Census tracts were used to define neighborhoods. Census tracts were chosen as the neighborhood unit given that they represent relatively homogenous units with respect to population characteristics, economic status, and living conditions. Census tracts generally range in population size between 1200 and 8000 people, with an optimum size of 4000 [34]. To construct the neighborhood-level built environment characteristics, we processed street imagery and then combined information from all street imagery within a census tract to create census tract-level summaries (e.g., the percentage of images in a census tract that contain visible wires).

### 2.3. Neighborhood Characteristics and Image Processing

The neighborhood characteristics examined included: (1) street greenness (street trees and street landscaping comprised at least 30% of an image (yes/no), (2) visible utility wires overhead (yes/no), and (3) dilapidated building (yes/no). These features of the built environment were selected based on prior review of the literature and the feasibility of implementation with computer vision models.

We operationalized street greenness as street trees and street landscaping comprising at least 30% of the image. A cut-point of approximately 30% was utilized to assist with inter-rater reliability in manual annotations of street greenness. Moreover, we found that most images had some street landscaping and therefore aimed to create a neighborhood indicator to distinguish between ample versus sparse street landscaping.

In this study, images with dilapidated buildings and visible utility wires overhead were utilized as indicators of physical disorder. Dilapidated buildings and vacant lots represent visible cues of physical disorder in the neighborhood environment and have been associated with adverse physical and mental health [18,35].

### 2.4. Image Data Processing

Images were processed using trained Visual Geometry Group (VGG-19 model) deep convolutional networks (previously detailed by Nguyen et al. [36,37]) to identify the built environment features of interest (one network per feature). Briefly, Convolutional Neural Networks (ConvNets) [38,39,40] achieve state-of-the-art accuracy for several computer vision tasks including but not limited to object recognition, object detection, and scene labeling. For example, the state-of-the-art accuracy of ImageNet with 1000 categories and over one million image samples is improved every year using ConvNet-based methods. The ImageNet dataset contains images from various categories (e.g., “motorcycle”, “apple”) and corresponding category labels. Models trained on this dataset use trial and error to learn combinations of colors, shapes, and textures that are relevant to a wide variety of image interpretation tasks, and therefore can be used as a starting point for creating computer vision models for tasks where labeled training data are scarce. A ConvNet model “pre-trained” on ImageNet can be “fine-tuned” using a smaller amount of training data from the desired task, which delivers strong classification performance without requiring the vast training data and computational resources necessary to train the original ConvNet. For this study, we used the pre-trained ConvNet model with additional training data from our research team to fine-tune the computer vision models to label GSV images for each specific built environment characteristic.

We randomly divided the GSV dataset into a training set, a validation set, and a test set. The training and validation set contained 80% of the total labeled images and the remaining 20% was used as a test set to evaluate the model’s performance. Once the hyper-parameters were chosen, each model architecture was trained multiple times. Note that neural network training is stochastic even when starting from the same initialization and using the same training set. Therefore, multiple training runs are used to assess the mean and standard deviation of the error. The testing set remained unobserved until the best models had been selected using the training set. We assessed the final quality of the model using the test set.

To create a training dataset for our computer vision models of street greenness (trees and landscaping comprised at least 30% of the image) and visible utility wires, from December 2016 to February 2017, we manually annotated 18,700 GSV images (from Chicago, Illinois; Salt Lake City, Utah; Charleston, West Virginia; and a national sample). These locations were chosen to capture heterogeneity in neighborhood environments across geographically and visually distinct places with varying population densities, urban development, and demographics. The manually labeled data help the computer vision models learn to detect the built environment features we are interested in extracting from GSV images. Labelers included the principal investigator and three graduate research assistants. Inter-rater agreement was above 85% for all neighborhood characteristics.

Our training dataset for dilapidated buildings consisted of approximately 29,400 GSV images captured from Baltimore and Detroit based upon administrative lists from city governments on vacant buildings and buildings marked for demolition from 2014 to 2018. The dataset has an equal number of normal and dilapidated buildings.

For all three measures, we then trained a standard deep convolutional neural network architecture: ResNet-18 in Pytorch with NLL loss as the loss function. In the test set, accuracy of the recognition tasks (agreement between manually labeled images and computer vision predictions) was 88.7% for street greenness, 83.0% for visible utility wires, and 89.1% for dilapidated buildings. These figures were consistent with a separate, semi-supervised learning approach.

### 2.5. Individual-Level Health Outcome Data

We examined the association of measures of the built environment from GSV with patient health outcomes in a cross-sectional design. Our analytic data included electronic health records for UCSF Health patients aged 18 years and older who had at least two outpatient visits during a 2-year period from January 1, 2015 to December 31, 2017. We obtained data on health outcomes based on International Classification of Diseases, Clinical Modification codes (ICD-9-CM and ICD-10-CM) documented in an encounter, on the problem list, or within the medical history during or prior to the study period. We focused on chronic disease outcomes of coronary artery disease (ICD-9-CM 410.0-414.9; ICD-10-CM I20.0-I25.9), hypertension (ICD-9-CM 401.0-405.99; ICD-10-CM I10-I15.9), and diabetes (ICD-9-CM 250.x; ICD-10-CM E10.10-E13.9). To identify independent associations between the built environment and health outcomes, we adjusted for patient-level characteristics: self-reported age, gender (female, male), married/significant other (yes/no), and race/ethnicity (American Indian/Alaska Native, Asian, Black, Hispanic, Native Hawaiian or Pacific Islander, White, or Other), English as the patient’s preferred language (yes/no), insurance status (private/Medicare Advantage, Medicare, Medicaid/Medi-Cal, or unspecified/charity), and assignment of a primary care provider (yes/no). Insurance status, English as the patient’s preferred language, and assignment of a primary care provider were included as proxy measures of individual-level SES since direct measures (e.g., income, education) are not widely available in the EHR. We also accounted for neighborhood socioeconomic status (nSES) using a previously developed composite index created from principal components analysis of seven indicators based on the established nSES index developed by Yost and colleagues [41] as well as Yang and colleagues [42]. The index uses 2013-2017 American Community Survey 5-year rolling averages data on census tract-level income, education, poverty, employment, occupation, housing and rent values [43]. Quintiles of nSES were based on the distribution of nSES index values across all census tracts in California, with the first quintile reflecting the lowest nSES and the fifth quantile reflecting the highest nSES census tracts. Patients’ residential addresses from the EHR were geocoded to latitude and longitude coordinates using ArcGIS Business Analyst and then assigned a 2010 U.S. Census tract [44]. Approximately 96% of patients had an address in the EHR that could be geocoded.

### 2.6. Statistical Analyses

We conducted descriptive analyses of all patient characteristics including their geographic location, neighborhood environment features, and health outcomes. Patient electronic health records data were merged with neighborhood characteristics, including GSV-derived built environment characteristics and neighborhood SES, by census tract ID. We utilized logistic regression to examine associations between GSV-derived built environment features and individual health outcomes. To allow for nonlinear associations, GSV-derived built environment characteristics were categorized into tertiles based on the percentage of GSV images in each census tract with that feature, scaled to the distribution across all census tracts in California, with the first tertile (lowest) serving as the referent group. Models accounted for clustering at the census tract-level and adjusted for all patient characteristics (including nSES) described above. Separate regressions were run for each health outcome and each GSV-based environment feature examined. We performed sensitivity analyses where missing values for marital status, race/ethnicity, and smoking status were coded as an additional category rather than set to missing. The estimated main effects were very similar to the complete case analysis and are included in the Supplemental Materials (Table S1). In secondary analyses, we repeated all regressions stratified by race/ethnicity (Asian, Black, Hispanic/Latino, and White; other groups did not have sufficient numbers of patients). Residential segregation of racialized groups due to structural racism has resulted in differential neighborhood-level physical and social conditions and complex patterns between environmental factors, race/ethnicity and health, such that the association between environmental characteristics and health outcomes may not be equal across racial/ethnic groups [45]. Analyses were conducted using Stata MP 16.0 (StataCorp LP, College Station, TX, USA).

## 3. Results

Among 306,595 adult patients with at least two outpatient visits over the 2-year study period, 12,987 residing outside California, 60 missing covariate nSES information, and 79,385 missing patient-level covariate information were excluded for a final analytic sample of 214,163. Included patients resided in 60% of census tracts in California, including all Bay Area census tracts. Patients had a mean age of 53 years, 52% were married or had a significant other, 59% were white race/ethnicity and 57% were women (Table 1). The majority of patients had private insurance or Medicare (79%). Approximately 7% of patients were diagnosed with coronary artery disease, 28% were diagnosed with hypertension, and 12% with diabetes.

**Figure 1:**
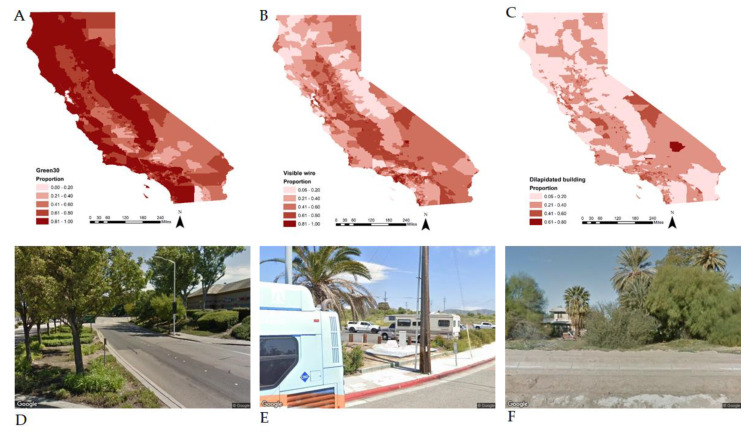
Spatial distribution of Google Street View (GSV)-derived built environment characteristics across census tracts in Cali-fornia. For each census tract, the following was calculated: percentage of GSV images with (**A**) Green30—street land-scaping comprising at least 30% of the image; (**B**) visible utility wires overhead; and (**C**) dilapidated buildings. Census tract characteristics were spatially mapped and darker colors represent higher values. Below the maps, sample GSV images are presented. The left panel gives an example of a green street (**D**), the second panel gives an example of visi-ble utility wires (**E**), and the third panel gives an example of dilapidated buildings (**F**). Data source: Google Street View images.

GSV data were available for all census tracts where patients resided. Figure 1 displays the spatial distribution of the three GSV-derived built environment characteristics across census tracts in California. High levels of green streets seem to be widely dispersed throughout California, whereas high levels of visible utility wires were less widely dispersed and generally concentrated in census tracts with fewer green streets. Dilapidated buildings seem to be concentrated in relatively few census tracts.

We examined associations between GSV built environment characteristics and individual health outcomes independent of neighborhood and individual-level SES and other covariates (Table 2). We found that higher proportions of streets with green space were associated with lower prevalence of coronary artery disease, hypertension, and diabetes. Specifically, patients living in census tracts in the third (highest) tertile of green streets had 26% lower odds of having coronary artery disease (95% CI: 0.71, 0.78) compared to residents living in census tracts in the lowest tertile. Higher proportions of visible wires and dilapidated buildings were associated with a greater prevalence of these chronic health outcomes. Patients living in census tracts in the highest tertile of visible wires had 21% higher odds of having coronary artery disease (95% CI: 1.14, 1.28), and patients living in census tracts in the highest tertile of dilapidated buildings had 18% higher odds of having coronary artery disease (95% CI: 1.11, 1.25) compared to patients living in census tracts in the lowest corresponding tertile. Similar associations were observed for hypertension and diabetes.

In exploratory analyses, we fitted race/ethnicity stratified models. Descriptive characteristics of the analytic sample by race/ethnicity are presented in the online appendix (Supplementary Material Table S2). Among racial/ethnic minority patients, associations tended to be in the same direction but attenuation compared to the non-stratified results, and some of these effect estimates were no longer statistically significant (Supplementary Material Table S3). However, there may not have been sufficient sample size for all racial/ethnic groups. For example, while there were approximately 125,494 White patients, there were 13,027 Black and 23,442 Hispanic patients in our analytic sample. Nonetheless, the association between green space and hypertension remained statistically significant for all racial/ethnic minority groups. Further, the association between dilapidated buildings and hypertension remained statistically significant for Black and Hispanic/Latino patients whereas the association between visible wires and hypertension remained statistically significant for Asian patients.

## 4. Discussion

This study found that the green streets were associated with a lower prevalence of coronary artery disease, hypertension, and diabetes, while visible utility wires and dilapidated buildings were generally associated with higher prevalence of these conditions after adjusting for patient sociodemographic characteristics and neighborhood SES. Our findings align with previous literature findings that neighborhood aesthetics such as greenery are associated with lower chronic medical conditions [18], whereas measures of neighborhood physical disorder are associated with worse health outcomes [14]. Physical disorder in the built environment is a sign of a lack of neighborhood investment and has been associated with an array of poor health outcomes including functional limitations, chronic health conditions, and poorer self-reported health, even when controlling for individual-level socioeconomic status [13,14,15]. Neighborhood physical disorder has also been related to reduced physical activity [16,17]. Similarly, neighborhood greenness has been associated with increases in physical activity, reduction in chronic health conditions, and improvements in mental health [18,19,20].

This is among the few studies examining the association of GSV-derived measures of environmental factors and individual-level health outcomes as opposed to neighborhood-level health outcomes. Previous studies with GSV images have utilized ecological frameworks with county- or census tract-level built environment predictors and health outcomes [20]. For example, a previous ecological study examined associations between census tract-level GSV built environment characteristics and census tract self-reported health outcomes from the 500 Cities Project [20]. Our study is a multilevel investigation assessing associations between census tract-level neighborhood built environment characteristics and individual health outcomes from electronic health records. UCSF Health provides outpatient care to over 350,000 individuals annually with patients residing throughout California.

Additionally, our study is among the few U.S. based studies that examine associations between visible utility wires and health outcomes. We found consistent associations between visible wires and all three chronic conditions. Given this is an understudied area, replication studies are needed as well as studies that explore the potential mechanisms operating. In race-specific analyses, a greater number of associations reached statistical significance among white patients compared to other racial/ethnic groups, but the number of white patients was also 4–10 times the number of patients of other groups, thereby also permitting greater statistical power. Race-specific investigations are less common but are further needed to investigate heterogeneity in effect estimates. Race-specific analyses suggest that built environment characteristics of green streets, visible utility wires, and dilapidated buildings are linked with cardiovascular outcomes, in particular hypertension. This aligns with other research connecting identifying chronic psychological stressors such as stressful aspects of the social and built environment and low socioeconomic status as risk factors for hypertension [46]. For example, analyses of the Multi-Ethnic Study of Atherosclerosis (MESA) found that lower neighborhood walkability, availability of healthy foods, perceived safety, and social cohesion were each associated with a greater likelihood of hypertension [47].

Our study is one of the first studies to demonstrate associations between GSV-derived built environment features and individual health outcomes independent of individual-level sociodemographic characteristics as well as a composite measure of neighborhood SES. We leveraged Google Street View images and computer vision to characterize neighborhood environments. However, other neighborhood characteristics not examined could also be important for chronic health conditions, including noise level, air pollution levels, and perceived safety of neighborhoods. For instance, previous studies have reported a positive association between perceived neighborhood safety and higher levels of walking activity [16].We did not include specific infrastructure such as bike lanes and transportation options in a neighborhood. Further, our study is subject to limitations. This study examined cross-sectional associations and cannot establish temporality between health outcomes and neighborhood exposure. Dates of changes in residential address are not available in the EHR, and in some cases disease onset may have preceded the most recent documented residential address of record or the GSV image to derive the neighborhood exposure. Further, results may not be generalizable to different patient populations. The analytic sample included patients from UCSF Health, was majority White (56%), and included patients that tended to live in higher SES neighborhoods. Sixty-nine percent of study participants resided in the two highest quintiles of neighborhood SES (scaled to all census tracts in California). Studies with larger samples of non-White patients would permit more thorough investigations of how associations between neighborhood built environment characteristics and health may differ by race/ethnicity. Additionally, using computer vision can limit the types and number of features that can be examined. Computer vision is more accurate for features that are relatively large in size, which makes detection easier. For instance, computer vision can more easily detect cars compared to litter. Moreover, the number of features that could be examined was limited given that training datasets were compiled from manual annotations of each neighborhood feature and separate computer vision models were built for each feature. Thus, unlike onsite neighborhood inventories that can consist of potentially hundreds of neighborhood features, we focus on a few core neighborhood features that have been theoretically and empirically connected to health outcomes. Additionally, although we adjusted for several important covariates, residual and unmeasured confounding may have persisted. In particular, measures of individual SES in the electronic health record were limited and likely did not fully account for variation in individual SES. Similarly, health outcomes were based on diagnoses in the electronic health record and may be subject to misclassification.

## 5. Conclusions

This study revealed that GSV features can be extracted by current computer vision models and can help us in predicting health outcomes above and beyond individual-level patient sociodemographic characteristics and a composite measure of neighborhood SES. Such environmental features include green landscaping, which was associated with a lower prevalence of coronary artery disease, hypertension, and diabetes, as well as features of physical disorder including visible utility wires and dilapidated buildings, which were found to be associated with a higher prevalence of chronic health conditions. Some healthcare systems collect information on their patients’ neighborhood SES (e.g., percent poverty). Fewer are tracking neighborhood built environment. Our study highlights to healthcare systems that neighborhood context may impact patient health, further underscoring the value of taking these characteristics into account when examining contributors to their patients’ health. GSV images and computer vision models can be used to understand contextual factors influencing patient health outcomes and inform structural and place-based interventions to promote population health. Interventions might include modifications of neighborhood environments to include more green space, street landscaping, and repairing buildings and infrastructure.

## Figures and Tables

**Table 1:** Sociodemographic characteristics of the sample (N = 214,163).

Characteristic	N (%)
Age (Mean, SD)	53.41 (17.93)
Female	121,829 (56.89)
Married/Significant Other	110,510 (51.60)
Insurance	
Private/Medicare Advantage	115,193 (53.79)
Medicare	53,864 (25.15)
Medicaid/Medi-Cal	28,643 (13.37)
Unspecified/Charity	16,463 (7.69)
Race/Ethnicity	
American Indian or Alaska Native	783 (0.37)
Asian	33,103 (15.46)
Black or African American	13,027 (6.08)
Hispanic/Latino	23,442 (10.95)
Native Hawaiian or Other Pacific Island	3331 (1.56)
Other	14,983 (7.00)
White or Caucasian	125,494 (58.60)
English preferred language	197,064 (92.02)
Smoking status	
Current smoker	14,682 (6.86)
Former smoker	55,156 (25.75)
Never smoker	144,325 (67.39)
Assigned primary care provider	172,818 (80.69)
Coronary artery disease	14,426 (6.74)
Hypertension	60,129 (28.08)
Diabetes mellitus	25,841 (12.07)
Neighborhood SES	
1st Quintile	13,127 (6.13)
2nd Quintile	19,705 (9.20)
3rd Quintile	30,773 (14.37)
4th Quintile	46,285 (21.61)
5th Quintile	104,273 (48.69)
Google Street View Built Environment	
Green space	
1st Tertile (lowest)	97,430 (45.49)
2nd Tertile	47,028 (21.96)
3rd Tertile	69,705 (32.55)
Visible wires	
1st Tertile (lowest)	76,371 (35.66)
2nd Tertile	65,214 (30.45)
3rd Tertile	72,578 (33.89)
Dilapidated buildings	
1st Tertile (lowest)	85,161 (39.76)
2nd Tertile	70,439 (32.89)
3rd Tertile	58,563 (27.35)

**Table 2:** Associations between Google Street View-derived built environment characteristics and coronary artery disease, hypertension, and diabetes (N = 214,163).

Characteristic (Higher Tertiles Indicate Higher Prevalence)	Coronary Artery Disease	Hypertension	Diabetes
	Prevalence Ratio (95% CI)	Prevalence Ratio (95% CI)	Prevalence Ratio (95% CI)
Green streets, 3rd tertile	0.74 (0.71, 0.78) *	0.71 (0.68, 0.74) *	0.84 (0.80, 0.88) *
Green streets, 2nd tertile	0.93 (0.88, 0.99) *	0.94 (0.90, 0.98) *	1.00 (0.95, 1.05)
Visible wires, 3rd tertile	1.21 (1.14, 1.28) *	1.24 (1.18, 1.32) *	1.10 (1.05, 1.16) *
Visible wires, 2nd tertile	1.13 (1.06, 1.19) *	1.19 (1.13, 1.25) *	1.09 (1.04, 1.15) *
Dilapidated building, 3rd tertile	1.18 (1.11, 1.25) *	1.19 (1.13, 1.25) *	1.14 (1.09, 1.20) *
Dilapidated building, 2nd tertile	1.15 (1.09, 1.22) *	1.18 (1.13, 1.24) *	1.15 (1.10, 1.21) *

Adjusted logistic regression specifying clustering at the census tract-level and controlling for the following covariates: age, sex, marital status, race/ethnicity, insurance status, English as preferred language, assignment of a primary care provider, and neighborhood SES index. The 1st (lowest) tertile of each characteristic is the referent (based on the percentage of Google Street View (GSV) images with the characteristic). * *p* < 0.05.

## Data Availability

The patient health dataset generated and/or analyzed in this study is not publicly available due to the personal and sensitive nature of included identifying information, including patient residential address, and the potential harm that could arise from sharing this information.

## Supplementary Materials

The following are available online at https://www.mdpi.com/article/10.3390/ijerph181910428/s1, Table S1: Associations between Google Street View-derived built environment characteristics and coronary artery disease, hypertension, and diabetes (N = 284,904), Table S2: Sociodemographic characteristics of the sample by race/ethnicity, Table S3: Associations between Google Street View-derived built environment characteristics and coronary artery disease, hypertension, and diabetes by race and ethnicity.
