# Supplementary material for: Google Street View-Derived Neighborhood Characteristics in California Associated with Coronary Heart Disease, Hypertension, Diabetes
Source: Int J Environ Res Public Health. 2021 Oct 3;18(19):10428. doi: 10.3390/ijerph181910428 (PMC8507846; doi:10.3390/ijerph181910428)
Supplement: Supplementary file 1 [file ijerph-18-10428-s001.zip › ijerph-1367890-supplementary.pdf]

Online Supplemental Materials

Table S1. Associations between Google Street View-derived built environment characteristics and coronary artery disease, hypertension, and diabetes (N=284,904).

	**Coronary artery disease**	**Hypertension**	**Diabetes**
	Prevalence Ratio (95% CI)	Prevalence Ratio (95% CI)	Prevalence Ratio (95% CI)
Characteristic (higher tertiles indicate higher prevalence)			
Green streets, 3rd tertile	0.73 (0.70, 0.77)*	0.68 (0.65, 0.71)*	0.84 (0.80, 0.88)*
Green streets, 2nd tertile	0.93 (0.88, 0.99)*	0.91 (0.87, 0.95)*	1.01 (0.96, 1.06)
Visible wires, 3rd tertile	1.23 (1.16, 1.30)*	1.31 (1.24, 1.38)*	1.10 (1.04, 1.15)*
Visible wires, 2nd tertile	1.14 (1.08, 1.20)*	1.21 (1.15, 1.28)*	1.10 (1.05, 1.16)*
Dilapidated building, 3rd tertile	1.18 (1.12, 1.25)*	1.21 (1.15, 1.28)*	1.14 (1.09, 1.20)*
Dilapidated building, 2nd tertile	1.17 (1.11, 1.23)*	1.21 (1.15, 1.26)*	1.16 (1.11, 1.21)*

Adjusted logistic regression specifying clustering at the census tract level and controlling for the following covariates: age, sex, marital status, race/ethnicity, insurance status, English as preferred language, assignment of a primary care provider, and neighborhood SES index; 1st (lowest) tertile of each characteristic is the referent (based on percentage of Google Street View (GSV) images with the characteristic). Missing values for marital status, race/ethnicity, and smoking status were coded as an additional category rather than set to missing in models.
*p<0.05

Table S2. Sociodemographic characteristics of the sample by race/ethnicity.

Characteristic	Asian N=33,103	Black (N=13,027)	Hispanic/Latino N=23,442	White N=125,494
Age (Mean, SD)	52.80 (18.73)	51.98 (17.09)	48.05 (17.52)	55.27 (17.56)
Female	20,621 (62.29)	7,623 (58.52)	13,793 (58.84)	68,890 (54.90)
Married/SignificantOther	19,104 (57.71)	3,407 (26.15)	10,791 (46.03)	67,907 (54.11)
Insurance				
Private/MedicareAdv	18,859 (56.97)	4,391 (33.71)	10,083 (43.01)	69,385 (55.29)
Medicare	6,979 (21.08)	3,486 (26.76)	4,446 (18.97)	35,391 (28.20)
Medicaid/Medi-cal	4,153 (12.55)	3,973 (30.50)	6,894 (29.41)	10,377 (8.27)
Workers' Comp	371 (1.12)	173 (1.33)	381 (1.63)	843 (0.67)
Govt/Tricare	132 (0.40)	196 (1.50)	146 (0.62)	789 (0.63)
Unspecified/Charity	2,609 (7.88)	808 (6.20)	1,492 (6.36)	8,709 (6.94)
English pref language	25,747 (77.78)	12,932 (99.27)	18,172 (77.52)	122,813 (97.86)
Smoking status				
Current smoker	1,391 (4.20)	2,158 (16.57)	1,384 (5.90)	8,292 (6.61)
Former smoker	5,794 (17.50)	3,679 (28.24)	5,295 (22.59)	36,277 (28.91)
Never smoker	25,918 (78.30)	7,190 (55.19)	16,763 (71.51)	80,925 (64.49)
Assigned primary care provider	29,168 (88.11)	10,754 (82.55)	16,762 (71.50)	100,261 (79.89)
Coronary Artery Disease	2,339 (7.07)	1,168 (8.97)	1,306 (5.57)	8,339 (6.64)
Hypertension	11,156 (33.70)	5,942 (45.61)	7,129 (30.41)	31,123 (24.80)
Diabetes Mellitus	5,424 (16.39)	2,641 (20.27)	4,345 (18.54)	10,900 (8.69)
Neighborhood SES				
1st Quintile	1,121 (3.39)	2,175 (16.70)	4,011 (17.11)	4,720 (3.76)
2nd Quintile	1,609(4.86)	2,110(16.20)	3,831(16.34)	10,642(8.48)
3rd Quintile	4,820(14.56)	2,417(18.55)	4,835(20.63)	15,957(12.72)
4th Quintile	8,389(25.34)	3,215(24.68)	4,812(20.53)	25,564(20.37)
5th Quintile	17,164(51.85)	3,110(23.87)	5,953(25.39)	68,611(54.67)
Google Street View indicators				
Green Space				
1st Tertile	22,042 (66.59)	7,099 (54.49)	11,652 (49.71)	46,803 (37.30)
2nd Tertile	5,725 (17.29)	3,912 (30.03)	6,313 (26.93)	26,856 (21.40)
3rd Tertile	5,336 (16.12)	2,016 (15.48)	5,477 (23.36)	51,835 (41.30)
Visible Wires				
1st Tertile	7,774 (23.48)	2,607 (20.01)	5,854 (24.97)	54,132 (43.14)
2nd Tertile	8,540 (25.80)	3,961 (30.41)	8,375 (35.73)	38,574 (30.74)
3rd Tertile	16,789 (50.72)	6,459 (49.58)	9,213 (39.30)	32,788 (26.13)
Dilapidated buildings				
1st Tertile	9,428 (28.48)	3,997 (30.68)	7,845 (33.47)	57,103 (45.50)
2nd Tertile	12,977 (39.20)	4,833 (37.10)	8,357 (35.65)	37,575 (29.94)
3rd Tertile	10,698 (32.32)	4,197 (32.22)	7,240 (30.88)	30,816 (24.56)

Table S3. Associations between Google Street View-derived built environment characteristics and coronary artery disease, hypertension, and diabetes by race and ethnicity.

	Coronary artery disease	Hypertension	Diabetes
	**Prevalence Ratio (95% CI)**	**Prevalence Ratio (95% CI)**	**Prevalence Ratio (95% CI)**
GSV built environment characteristics			
Among Black patients (N=13,027)			
Green streets, 3rd tertile	0.82 (0.67, 1.02)	0.77 (0.68, 0.88)*	0.92 (0.80, 1.07)
Green streets, 2nd tertile	0.94 (0.82, 1.07)	0.97 (0.89, 1.06)	0.98 (0.88, 1.09)
Visible wires, 3rd tertile	1.09 (0.92, 1.30)	1.07 (0.95, 1.20)	0.99 (0.88, 1.12)
Visible wires, 2nd tertile	1.12 (0.93, 1.35)	1.01 (0.89, 1.15)	1.00 (0.87, 1.15)
Dilapidated building, 3rd tertile	0.98 (0.84, 1.14)	1.08 (0.97, 1.20)	0.96 (0.85, 1.07)
Dilapidated building, 2nd tertile	0.98 (0.83, 1.15)	1.14 (1.04, 1.26)*	1.07 (0.96, 1.19)
Among Hispanic/Latino patients (N=23,442)			
Green streets, 3rd tertile	0.89 (0.76, 1.04)	0.78 (0.71, 0.85)*	0.88 (0.80, 0.97)
Green streets, 2nd tertile	1.13 (0.97, 1.32)	0.96 (0.88, 1.04)	1.00 (0.92, 1.10)
Visible wires, 3rd tertile	0.97 (0.82, 1.15)	1.12 (1.02, 1.23)*	1.03 (0.93, 1.14)
Visible wires, 2nd tertile	0.87 (0.74, 1.03)	1.04 (0.95, 1.14)	1.06 (0.95, 1.17)
Dilapidated building, 3rd tertile	1.08 (0.93, 1.27)	1.14 (1.05, 1.24)*	1.13 (1.02, 1.24)*
Dilapidated building, 2nd tertile	1.15 (0.99, 1.34)	1.14 (1.05, 1.25)*	1.06 (0.97, 1.16)
Among Asian patients (N=33,103)			
Green streets, 3rd tertile	0.77 (0.66, 0.90)*	0.69 (0.62, 0.77)*	0.86 (0.77, 0.96)*
Green streets, 2nd tertile	0.97 (0.85, 1.11)	1.00 (0.92, 1.10)	1.06 (0.96, 1.18)
Visible wires, 3rd tertile	1.09 (0.95 , 1.24)	1.19 (1.06, 1.34)*	0.98 (0.88, 1.09)
Visible wires, 2nd tertile	1.10 (0.94, 1.27)	1.25 (1.12, 1.40)*	1.02 (0.91, 1.14)
Dilapidated building, 3rd tertile	1.10 (0.98, 1.24)	1.07 (0.95, 1.20)	1.03 (0.93, 1.15)
Dilapidated building, 2nd tertile	1.06 (0.94, 1.19)	1.05 (0.96, 1.16)	1.04 (0.94, 1.14)
Among White patients (N=125,494)			
Green streets, 3rd tertile	0.73 (0.69, 0.78)*	0.72 (0.69, 0.76)*	0.89 (0.83, 0.95)*
Green streets, 2nd tertile	0.88 (0.81, 0.95)*	0.92 (0.87, 0.98)*	1.01 (0.95, 1.09)
Visible wires, 3rd tertile	1.29 (1.19, 1.39)*	1.27 (1.20, 1.35)*	1.12 (1.05, 1.19)*
Visible wires, 2nd tertile	1.14 (1.07, 1.22)*	1.20 (1.13, 1.26)*	1.07 (1.01, 1.14) *
Dilapidated building, 3rd tertile	1.15 (1.05, 1.27)*	1.20 (1.13, 1.27)*	1.16 (1.09, 1.24)*
Dilapidated building, 2nd tertile	1.15 (1.07, 1.24)*	1.20 (1.14, 1.27)*	1.19 (1.12, 1.26)*

Adjusted logistic regression specifying clustering at the census tract level and controlling for the following covariates: age, sex, marital status, race/ethnicity, insurance status, English as preferred language, and assignment of primary care provider, and neighborhood SES quintiles; 1st (lowest) tertile of each GSV indicator is the referent. *p<0.05
